# Hepato‐splanchnic fluxes during exercise in patients with cirrhosis—a pilot study

**DOI:** 10.14814/phy2.16162

**Published:** 2024-09-25

**Authors:** Stefanos Volianitis, Niels H. Secher, Otto Clemmesen, Peter Ott, Henning Bay Nielsen

**Affiliations:** ^1^ Department of Physical Education, College of Education Qatar University Doha Qatar; ^2^ Department of Anesthesia, Rigshospitalet University of Copenhagen Copenhagen Denmark; ^3^ Department of Clinical Medicine University of Copenhagen Copenhagen Denmark; ^4^ Department of Transplantation and Digestive Diseases, Section for Intestinal Failure and Liver Diseases, Rigshospitalet University of Copenhagen Copenhagen Denmark; ^5^ Department of Hepatology and Gastroenterology Aarhus University Hospital Aarhus Denmark; ^6^ Department of Anesthesia and Intensive Care, Zealand University Hospital Roskilde University of Copenhagen Copenhagen Denmark

**Keywords:** ammonia uptake, glucose release, lactate elimination, liver blood flow, oxygen uptake

## Abstract

In cirrhotic patients, compromised hepatocyte function combined with disturbed hepatic blood flow could affect hepato‐splanchnic substrate and metabolite fluxes and exacerbate fatigue during exercise. Eight cirrhotic patients performed incremental cycling trials (3 × 10 min; at light (28 [19–37] W; median with range), moderate (55 [41–69] W), and vigorous (76 [50–102] W) intensity). Heart rate increased from 68 (62–74) at rest to 95 (90–100), 114 (108–120), and 140 (134–146) beats/min (*P* < 0.05), respectively. The hepatic blood flow, as determined by constant infusion of indocyanine green with arterial and hepatic venous sampling, declined from 1.01 (0.75–1.27) to 0.69 (0.47–0.91) L/min (*P* < 0.05). Hepatic glucose output increased from 0.6 (0.5–0.7) to 1.5 (1.3–1.7) mmol/min, while arterial lactate increased from 0.8 (0.7–0.9) to 9.0 (8.1–9.9) mmol/L (*P* < 0.05) despite a rise in hepatic lactate uptake. Arterial ammonia increased in parallel to lactate from 47.3 (40.1–54.5) to 144.4 (120.5–168.3) μmol/L (*P* < 0.05), although hepatic ammonia uptake increased from 19.5 (12.4–26.6) to 69.5 (46.5–92.5) μmol/min (*P* < 0.05). Among the 14 amino acids measured, glutamate was released in the liver, while the uptake of free fatty acids decreased. During exercise at relatively low workloads, arterial lactate and ammonia levels were comparable to those seen in healthy subjects at higher workloads, while euglycemia was maintained due to sufficient hepatic glucose production. The accumulation of lactate and ammonia may contribute to exercise intolerance in patients with cirrhosis.

## 1 INTRODUCTION

Exercise poses a unique challenge to the liver which is required to mobilize energy stores, recycle metabolites, and convert toxic compounds to harmless forms (Trefts et al., 2015). In patients with cirrhosis of the liver, aerobic exercise capacity is limited compared to healthy controls (Jones et al., 2012) with detrimental consequences for both quality and expectancy of life (West et al., 2021). Factors that may contribute to impaired exercise tolerance in cirrhotic patients are low liver glycogen stores (Krähenbühl et al., 2003; Owen et al., 1981), combined with lactacidemia (Casaburi & Oi, 1989) and hypperammonemia (Dietrich et al., 1990) developed during exercise (Campillo et al., 1990; Epstein et al., 1998).

Theoretically, reduced liver glycogen stores combined with compromised glucagon‐induced glycogenolysis during exercise could reduce hepatic glucose production (Owen et al., 1983), which in turn could compromise glucose homeostasis and precipitate exercise‐induced hypoglycemia. This was suggested by De Lissio et al (1991). even though the cirrhotic patients in that study did not experience hypoglycemia during moderate, albeit prolonged exercise. As the De Lissio study employed indirect calorimetry and did not directly determine hepatic glucose output, the conclusion of compromised hepatic glucose production during exercise in cirrhotic patients is not definitive.

During exercise in healthy persons, there is a net‐uptake of lactate in the liver (Ahlborg et al., 1974; Rowell et al., 1966) that provides substrate for gluconeogenesis and accounts for ~20%–30% of lactate clearance (Stanley et al., 1988; van Hall, 2010). Compromised hepatic lactate clearance could precipitate arterial lactate accumulation and lower plasma pH with adverse metabolic and functional consequences for exercise capacity in cirrhosis (Casaburi & Oi, 1989; Nalbandian & Takeda, 2016). Yet, it is unclear whether hepatic lactate clearance is reduced in cirrhosis. In resting and fasting cirrhotic patients, evaluated with measurements in arterial and hepatic venous blood, arterial lactate was reported above normal (Jeppesen et al., 2013); however, as the hepatic clearance of lactate (removal rate/arterial concentration) was slightly higher in patients than in controls, this was more likely due to increased extrahepatic lactate production. Furthermore, exercise conditions were not studied (Jeppesen et al., 2013). Similarly inconclusive results are reports that infer impairment of hepatic lactate uptake in cirrhotic patients from prolonged lactate elimination half‐life, compared with healthy aged‐matched subjects, either in upper extremity venous or arterial plasma following lactate infusion (Woll & Record, 1979), or maximal exercise (Almenoff et al., 1989; Casaburi & Oi, 1989). Considering that other tissues including muscle, heart, brain, and kidneys are involved in lactate metabolism (Brooks et al., 2022), the specific contribution of hepatic lactate uptake to lactacidemia during exercise in cirrhotic patients remains to be elucidated.

Ammonia is produced by the metabolism of amino acids and proteins (Parnas, 1929) and is removed by the healthy liver (Yang et al., 2000) and other organs, including muscles. Vigorous exercise increases ammonia production in the muscles from which ammonia is released and, combined with a reduction in mesenteric blood flow (Qamar & Read, 1987), leads to increased plasma ammonia (Schwartz et al., 1958). Accumulation of ammonia is considered both a central and peripheral factor for exercise‐induced fatigue (Banister & Cameron, 1990; Parnas, 1929). In cirrhotic patients, the hypperammonemia developed during exercise was more pronounced than in healthy controls at similar relative workload and was attributed partially to decreased hepatic clearance, consequent to impaired hepatic function (Dietrich et al., 1990). However, in that study compromised hepatic ammonia uptake was deduced from delayed return to baseline of ante‐cubital venous plasma ammonia values following exercise (Dietrich et al., 1990). Considering that other tissues including muscle, brain, gut, and kidneys are contributing to ammonia metabolism, the part of hepatic ammonia removal to exercise‐induced hypperammonemia in cirrhotic patients also remains to be elucidated (Walker, 2012).

Only few data on the specific evaluation of liver metabolism with blood sampling from the hepatic vein during exercise are available for patients with cirrhosis. We collected blood samples from the hepatic vein and the brachial artery and calculated the arterial‐to‐hepatic vein differences and, in combination with measurements of hepato‐splanchnic (HS) blood flow, we assessed the HS fluxes for glucose, lactate, ammonia, amino acids, and free fatty acids (FFA) in compensated cirrhotic patients during exercise. We hypothesized that compromised hepatic substrate exchange may exacerbate metabolic factors that are important for exercise intolerance. Thus, this study aimed to provide a clearer picture of exercise‐induced changes in the hepatic release or uptake of metabolites, thereby improving understanding of the specific hepatic contribution to exercise metabolism and impaired exercise tolerance in cirrhosis.

## 2 MATERIALS AND METHODS

Eight patients with liver cirrhosis (Child Pugh class B, Table 1) were identified before routine measurement of portal pressure. Written informed consent was obtained from each patient included in the study, which conformed to the ethical guidelines of the 1975 Declaration of Helsinki and was approved a priori by the Copenhagen Ethical Committee (KF 01–303/00). Patients were included in the study if they were not in hepatic coma and presented with coagulation variables within the normal range to allow for invasive procedures. A control group was not included for ethical reasons because of the invasive nature of the procedures. Cirrhosis was related to hepatitis C, primary biliary cirrhosis, autoimmune hepatitis, Wilson's disease, or alcohol consumption. Three patients were receiving medication (either a beta‐adrenergic receptor blocker (two patients) or an angiotensin II receptor antagonist). The blood albumin concentration was 31 (27–35) g/L (normal range 37–48 g/L). The galactose elimination capacity (GEC) determined at least 8 h prior to the study was 20 (18–22) mmol/min/kg (normal range 30–35 mmol/min/kg (Tygstrup, 1964)) indicating reduced liver function. None of the patients had pulmonary or cardiac diseases.

**TABLE 1:** Anthropometric and clinical data.

	Gender (F/M)	Age (years)	Alb (g/l)	Bilirubin (μmol/l)	GEC mmol/min/kg	HVPG (mmHg)	Med
1	M	38	26	11	29	?	–
2	M	54	30	27	18	22	–
3	M	51	29	19	20	25	–
4	M	56	36	18	20	17	ACE
5	F	55	44	11	23	15	Beta
6	F	34	34	15	18	20	–
7	M	56	33	11	15	10	Beta
8	F	66	29	44	?	10	–

Abbreviations: Alb, albumin; GEC, galactose eliminating capacity; HVPG, hepatic venous pressure gradient; Med, medication.

The patients were studied after an overnight fast of 10 to 12 h and, considering that beta‐adrenergic blockade affects hepatic blood flow (Ahlborg & Juhlin‐Dannfelt, 1994), administration of vasoactive drugs was discontinued 24 h before the study. The patients were monitored throughout the protocol, and medical treatment was re‐commenced after the study. A brachial artery catheter (1 mm i. d.; 20 gauge) was inserted in the non‐dominant arm, while a liver venous catheter was introduced via the right median cubital vein and its position confirmed with fluoroscopy (Febbraio et al., 2003; Nielsen et al., 2002). The catheters were kept patent by continuous infusion of isotonic saline (3 ml h^−1^) and connected to a pressure monitoring kit (Baxter Healthcare Corporation, Maurepas, France) positioned at the level of the heart. The hepatic venous pressure gradient (HVPG) was calculated at rest as the difference between wedged and free hepatic venous pressures.

Exercise was performed on a semi‐recumbent cycle ergometer at 60 rpm (Febbraio et al., 2003; Nielsen et al., 2002) with light, moderate, and vigorous intensities (10 min at each workload). During the initial minute of each intensity, the workload was adjusted to a target heart rate of 90–95, 115–120, and 140 beats/min, respectively.

### 2.1 HS blood flow

The ICG (Cardio‐Green; Becton Dickinson, Cockeysville, MO; 0.18 (0.16–0.20) μmol/L) was infused into an arm vein (Ott et al., 1993) and, following a 45 min priming infusion to ensure a steady‐state plasma concentration, arterial and hepatic venous blood was collected simultaneously at 1 mL/s. Blood was sampled at rest, every third min in each workload, and if patients indicated that they were about to stop, at 5, 10, 15, 25, 35, and 45 min after exercise. Heparinised syringes (QS50; Radiometer, Copenhagen, Denmark) were kept in ice water until analysis for blood gas variables, glucose, lactate (ABL 615; Radiometer), ammonia, amino acids, and FFA.

Plasma was frozen at −20°C and the ICG concentration was determined using high‐performance liquid chromatography (Ott et al., 1993) and hepatic blood flow was calculated (Febbraio et al., 2003; Nielsen et al., 2002). Hepatic substrate exchange in each workload was the product of the average hepatic blood flow and the arteriovenous concentration difference of the substrate of interest. Heart rate and mean arterial pressure were assessed invasively (Baxter) and cardiac output estimated from the arterial pressure curve with Finometer® PRO, which uses the patented Modelflow® method (Wesseling et al., 1993) to provide hemodynamic parameters (Jansen et al., 2001). Pulmonary gas exchange was measured with an Oxyscreen (CPX/D; Medical Graphics Corporation, St. Paul, Minnesota) metabolic cart and 15 s averaged values reported. Glucose and FFA were determined using automated analyses (Cobas Fara, Roche, France) (Steensberg et al., 2001, 2002). We used the STROBE cross‐sectional checklist when writing our report (von Elm et al., 2008).

### 2.2 Ammonia

Ammonia was enzymatically measured in arterial and hepatic venous blood before exercise, at the end of the second and third workloads, and at the 25th and 45th minutes in the recovery period. One milliliter of blood was collected in a chilled, sterilized tube containing 15 IE of lithium heparin and was placed on ice immediately afterwards. Samples were centrifuged at 5°C and analyzed within 30 mins by use of a Kodak Ektachem 700 Analyzer, Clinical Chemistry Slide [HN3/ AMON] (Eastman Kodak Co., Rochester, NY).

### 2.3 Amino acids

For amino acids, plasma samples drawn at rest and at the end of each workload were deproteinized with 0.6 M perchloric acid (PCA; 1:5) and centrifuged at 9000 *g* for 2 min, and the supernatant was stored at −80°C until analysis. The concentration of 14 amino acids was measured by reversed‐phase HPLC as described by Pfeifer et al. (1983). with orthophthalaldehyde (OPA) as the derivatizing agent.

### 2.4 Statistics

Data are expressed as means with 95% CIs unless otherwise stated. Friedman analysis of variance (Stata v.14.2, StataCorp, CollegeStation, TX) and non‐parametric paired Wilcoxon rank test were used for comparisons among samples and across time. Sample size was guided by power calculations with SD and sample means of hepato‐splanchnic blood flow previously reported (Nielsen et al., 2002). With SD of 0.1, the sample size of *n* = 8 patients provided sufficient power (0.80) to detect a mean reduction in blood flow of 0.14 L/min. A *P*‐value below 0.05 was considered statistically significant.

## 3 RESULTS

Light‐, moderate‐, and vigorous‐intensity cycling were observed at 28 (19–37), 55 (41–69) and 76 (50–102) W, respectively. With VO_2_ at the highest workload considered to represent VO_2_peak, the light and moderate workloads corresponded to 54 (51–57) % and 78 (75–81) % of VO_2_peak, respectively. All patients completed the first two workloads and seven patients attempted the third workload: three patients (# 2, 4, and 7) cycled for 10 min, patients # 5 and # 6 were exhausted after 6 min, and patients # 1 and # 3 were exhausted after 3 min. Patient # 8 was exhausted at the end of the second workload.

Exercise increased heart rate, mean arterial pressure, cardiac output, and VO_2_ (*P* < 0.05, Table 2). After exercise, heart rate and cardiac output declined rapidly and mean arterial pressure was reduced to below the resting level.

**TABLE 2:** Circulatory and blood variables at rest and in response to incremental cycling in patients with liver cirrhosis.

	Rest	Exercise (min)			Recovery (min)					
		10	20	30	5	10	15	25	35	45
Systemic observations										
HR (beats/min)	68 (62–74)	95 (90–100)*	114 (108–120)*	140 (134–146)*	97 (92–102)*	92 (88–96)*	91 (88–94)*	88 (85–91)*	84 (81–87)	82 (80–84)
MAP (mmHg)	89 (86–92)	102 (96–108)*	109 (104–114)*	113 (108–118)*	84 (81–87)*	82 (78–86)*	80 (76–84)*	82 (78–86)*	81 (78–84)*	80 (77–83)
CO (L/min)	5.3 (4.8–5.8)	8.0 (7.2–8.8) *	10.0 (8.7–11.3)*	12.7 (11.4–14.0)*	7.5 (6.4–8.6)*	6.7 (5.9–7.5)	6.2 (5.6–7.0)	5.8 (5.2–6.4)	5.7 (5.2–6.2)	5.8 (5.3–6.3)
VO_2_ (L/min)	0.27 (0.25–0.29)	0.71 (0.50–0.92)*	1.16 (0.96–1.36)*	1.51 (1.29–1.73)*	0.32 (0.30–0.34)*	0.26 (0.24–0.28)	0.26 (0.24–0.28)	0.23 (0.21–0.25)	0.25 (0.23–0.27)	0.26 (0.24–0.28)
FFA (arterial, mM)	0.73 (0.68–0.78)		0.47 (0.44–0.50)	0.52 (0.39–0.65)			0.54 (0.45–0.63)			0.50 (0.39–0.61)
Hepato‐splanchnic										
BF (L/min)	1.01 (0.75–1.27)	0.84 (0.61–1.07)*	0.76 (0.49–1.03)*	0.69 (0.47–0.91)*	0.62 (0.38–0.66)*	0.66 (0.44–0.88)*	0.72 (0.48–0.96)	0.79 (0.57–1.01)	0.83 (0.60–1.06)	0.93 (0.67–1.19)
VO_2_ (ml/min)	48 (41–55)	50 (42–58)	55 (47–63)	66 (55–77)	50 (42–58)	41 (36–46)	45 (40–50)	44 (38–50)	44 (40–47)	45 (39–51)
Substrate exchange (−, uptake)										
FFA (mmol/min)	−0.26 (0.24–0.28)		−0.12 (011–0.13)*	−0.11 (0.08–0.14)*			−0.13 (0.10–0.16)*			−0.13 (0.10–0.16)*

*Note*: Values are median (range).

Abbreviations: BF, hepato‐splanchnic blood flow; CO, cardiac output; FFA, free fatty acids; HR, heart rate; MAP, mean arterial pressure; VO_2_, oxygen uptake; *, different from rest (*P* < 0.05).

**FIGURE 1:**
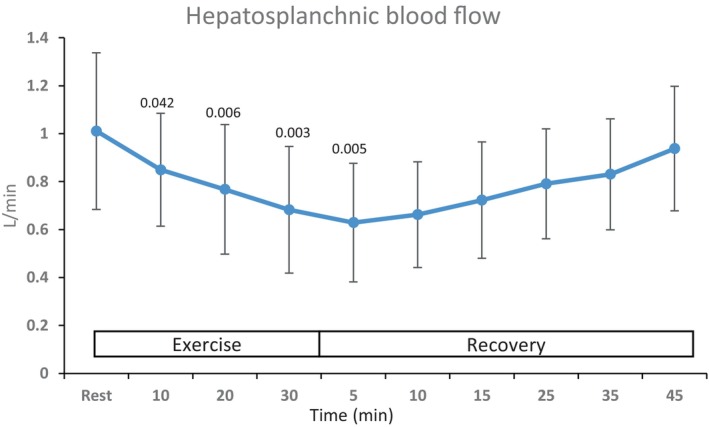
HS blood flow at rest, during mild (10 min), moderate (20 min), and vigorous (30 min) exercise and in the recovery. Values are mean (±SD). Numbers above means are p values for significant difference from rest.

The HVPG was 17 (10–25) mmHg, compared to a reference value of <5 mmHg. Hepato‐splanchnic blood flow (median [range]) was reduced by 13 [4–28] %, 23 [10–34] %, and 34 [14–46] % during light, moderate, and vigorous exercise, respectively, reaching a nadir 5 min after exercise (*P* < 0.05) (Figure 1; Table 2). Thereafter, HS blood flow increased to attain the pre‐exercise level after about 1 h.

### 3.1 Glucose flux

**FIGURE 2:**
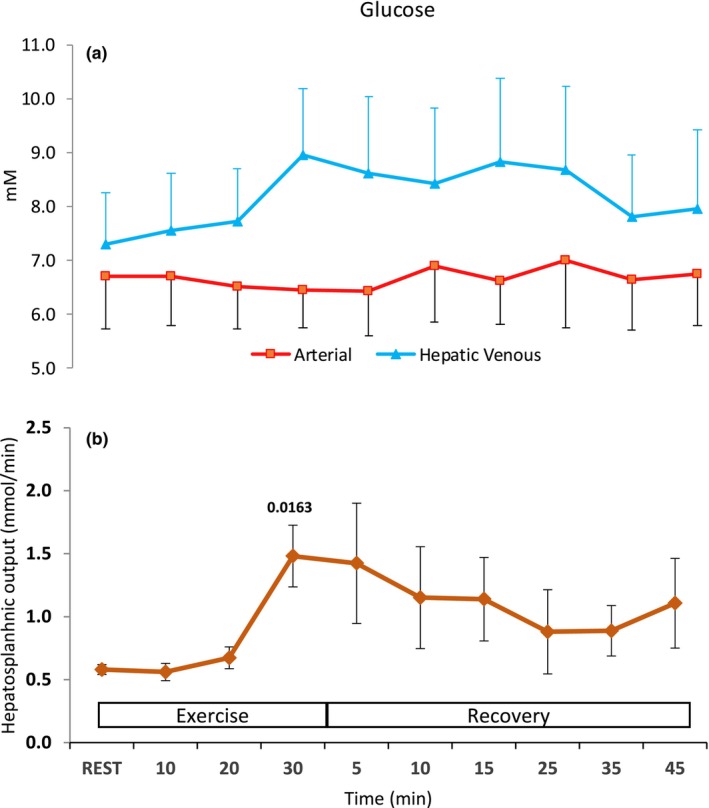
Glucose at rest, during mild (10 min), moderate (20 min), and vigorous (30 min) exercise and in the recovery. (a) Arterial and hepatic venous and (b) HS output. Values are mean (±SD). Numbers above means are *P*‐values for significant difference from rest.

Arterial glucose remained stable throughout the experimental period (Figure 2a). There was no change in the hepato‐splanchnic output of glucose during mild exercise compared to rest, whereas during moderate and vigorous exercise, it increased by ~25 [2–42] % and ~103 [60–419] %, respectively (*P* < 0.05, Figure 2b). The elevated glucose output was maintained at least ~50% higher than at rest throughout the recovery period (*P* < 0.05).

### 3.2 Lactate flux

**FIGURE 3:**
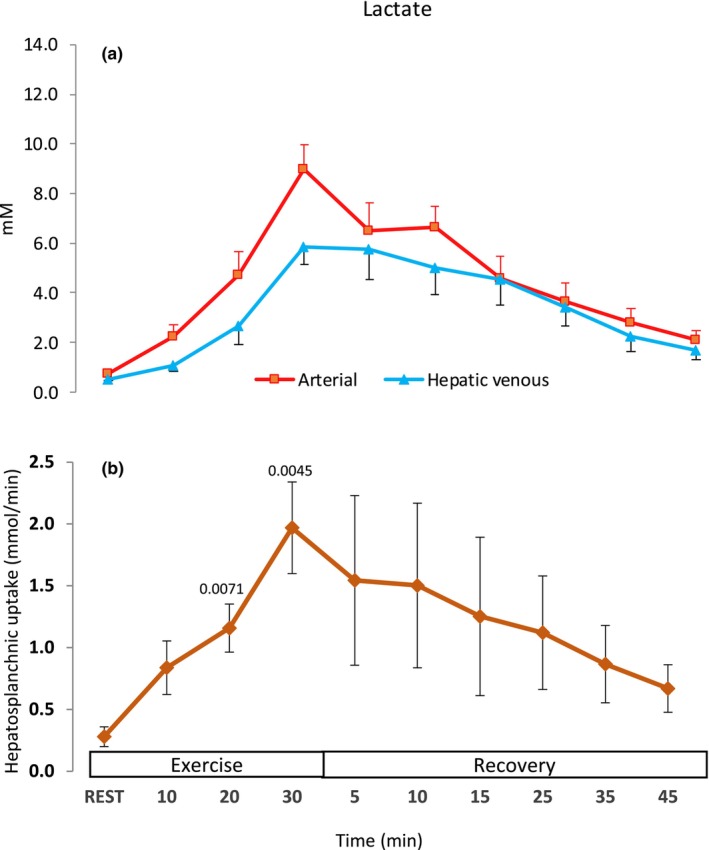
Lactate at rest, during mild (10 min), moderate (20 min), and vigorous (30 min) exercise and in the recovery. (a) Arterial and hepatic venous and (b) HS uptake. Values are mean (±SD). Numbers above means are p values for significant difference from rest.

Arterial lactate accumulation reached 9 mM at the end of vigorous exercise (*P* < 0.05, Figure 3a). During mild exercise, hepatic lactate uptake was tripled compared to that at rest and it was further increased four‐ and seven‐fold compared to that at rest during moderate and vigorous exercise, respectively (*P* < 0.05, Figure 3b). The time required for arterial lactate to return to half its maximal concentration (half‐time) was 15.1 (14.3–15.9) min during recovery.

### 3.3 Ammonia flux

**FIGURE 4:**
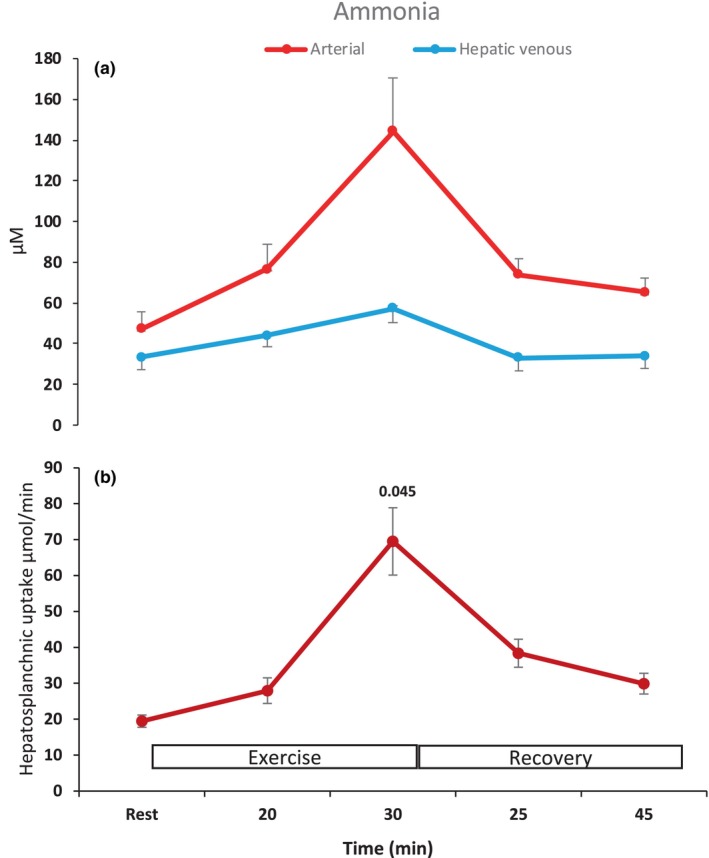
Ammonia at rest, during moderate (20 min) and vigorous (30 min) exercise, and in the recovery. (a) Arterial and hepatic venous, and (b) HS uptake. Values are mean (±SD). Numbers above means are *P*‐values for significant difference from rest.

At rest, arterial plasma ammonia was 47.3 (40.1–54.5) μM (Figure 4a). During moderate and vigorous exercise, hepatic ammonia uptake was increased by ~70 (64–76) % and more than tripled (~350 [338–374] %), respectively, compared to that at rest (*P* < 0.05, Figure 4b). However, arterial hyperammonemia was not prevented, as ammonia reached 144.4 (120.5–168.3) μM at the end of vigorous exercise (*P* < 0.05). The half‐time of arterial ammonia was 21.1 (20.0–22.2) min during recovery.

### 3.4 Free fatty and amino acids fluxes

The hepatic uptake of free fatty acids decreased both during exercise and recovery (Table 2). Among the amino acids measured (Table 3), the liver released glutamate during mild exercise; however, this output gradually returned to resting level by the end of the exercise trials. Glutamine was removed by the hepato‐splanchnic organs with no relation to exercise, and alanine uptake was constant.

**TABLE 3:** Concentrations of amino acids in arterial and hepatic venous blood and hepato‐splanchnic uptake of amino acids at rest and during light, moderate, and vigorous exercise.

		Rest			Light			Moderate			Vigorous	
	A	V	Uptake	A	V	Uptake	A	V	Uptake	A	V	Uptake
TAU	39 ± 4	39 ± 4	3.4 ± 0.6	43 ± 4	45 ± 4*	1.1 ± 1.9	43 ± 3	43 ± 3	−0.7 ± 0.4	38 ± 4	39 ± 4	−0.6
PEA	1.5 ± 0.3	1.7 ± 0.3	−0.2 ± 02	2.3 ± 0.4	1.6 ± 0.3	0.4 ± 0.3	1.9 ± 0.2	1.4 ± 0.2	0.3 ± 0.3	1.7 ± 0.2	1.6 ± 0.1	0.1 ± 0.1
OHPRO	6.0 ± 1.3	5.0 ± 1.6	0.5 ± 0.5	6.4 ± 1.6	4.1 ± 0.5	2.3 ± 1.2	6.2 ± 1.0	4.5 ± 0.7	1.0 ± 0.4	5.8 ± 1.2	4.4 ± 1.1	0.7 ± 0.1
THR	117 ± 9	98 ± 10	18.9 ± 3.0	118 ± 8	93 ± 9	24.3 ± 3.6	115 ± 8	91 ± 9•	20.7 ± 3.6	113 ± 8	93 ± 9•	15.6 ± 3.3
ASP	4.2 ± 0.8	5.7 ± 0.4	−2.5 ± 0.7	4.0 ± 0.9	6.7 ± 1.2	−3.1 ± 1.0	4.4 ± 1.1	6.1 ± 0.6	−2.1 ± 0.3	4.3 ± 1.1	4.8 ± 0.3	−1.1 ± 0.7*
SER	102 ± 6	84 ± 6	18.1 ± 5 0.1	96 ± 7*	73 ± 5*	23.7 ± 4.9	95 ± 7*	72 ± 5*	21.1 ± 4.8	96 ± 6*	75 ± 4*	17.1 ± 4.5
ASN	98 ± 10	67 ± 9	32.5 ± 4.7	107 ± 11*	69 ± 8	38.7 ± 5.9	101 ± 9	68 ± 8	30.6 ± 5.5	98 ± 10	70 ± 8	23.6 ± 5.5•
GLU	51 ± 13	137 ± 15	−78 ± 6	36 ± 9*	190 ± 23*	−131 ± 16*	49 ± 11	162 ± 18*	−81 ± 8	49 ± 9	138 ± 13	−57 ± 8*
GLN	587 ± 54	500 ± 42	71.9 ± 14.3	658 ± 51*	542 ± 44	90.1 ± 16.5	619 ± 39*	491 ± 39	86.9 ± 12.6	625 ± 51*	523 ± 42	68.8 ± 10.9
PRO	178 ± 16	160 ± 19	25.0 ± 5.5	180 ± 18	156 ± 18	28.8 ± 3.7	172 ± 18	155 ± 18	22.5 ± 6.0	163 ± 19	150 ± 23	10.7 ± 3.5
GLY	194 ± 5	172 ± 7	22.2 ± 7.8	206 ± 6*	183 ± 6	23.5 ± 7.6	189 ± 6	160 ± 8*	25.6 ± 7.6	188 ± 5	164 ± 8	19.8 ± 6.2
ALA	218 ± 11	127 ± 12	94.5 ± 9.8	380 ± 23*	288 ± 29*	84.7 ± 14.0	375 ± 29*	257 ± 41*	88.7 ± 13.1	332 ± 29*	204 ± 31*	89.9 ± 14.1
CTT	45 ± 3	53 ± 5	−10.6 ± 5.2	45 ± 3	48 ± 4	−2.6 ± 3.2	42 ± 3	46 ± 3	−3.1 ± 3.I	42 ± 2	49 ± 5	−6.0 ± 2.0
ABA	21 ± 3	21 ± 3	0.7 ± 0.9	20 ± 3	21 ± 3	0.4 ± 0.4	20 ± 3	21 ± 3	0.4 ± 0.6	20 ± 3	21 ± 3	−0.3 ± 1.0

*Note*: Values are mean ± SD. Concentrations are in μM and exchange rates in μmol/min.

Abbreviations: ABA, alpha‐amino‐n‐butyric acid; ALA, alanine; ASN, asparagine; ASP, aspartate; CTT, leucine; GLN, glutamine; GLU, glutamate; GLY, glycine; PEA, palmitoylethanolamide; PRO, proline; OHPRO, hydroxyproline; SER, serine; TAU, taurine; THR, threonine; *, different from rest (*P* < 0.05).

## 4 DISCUSSION

Hepato‐splanchnic fluxes of selected metabolites during incremental exercise in patients with cirrhosis were evaluated using blood samples from the hepatic vein and brachial artery. Vigorous exercise was associated with an elevated heart rate, cardiac output, and oxygen uptake, while HS blood flow was reduced by ~34%. Hepatic glucose output and lactate and ammonia uptake increased during exercise in patients with cirrhosis.

### 4.1 Exercise intensity

Although the adopted exercise protocol was not intended to be exhaustive, four patients (two males) were exhausted at different times during the vigorous workload indicating that they had reached their exercise capacity. The male patients achieved only ~40%, while the female only ~67% of their expected VO_2_max and associated workload compared with reference values of healthy people matched for age and sex (Loe et al., 2013). On average, the cirrhotic patients reached exhaustion, and associated high exercise metabolites (i.e., lactate and ammonia) with only half of the expected reference workload of healthy subjects. These data are consistent with the general observation of reduced exercise capacity in patients with cirrhosis (Jones et al., 2012).

### 4.2 Splanchnic circulation

As reported by Garcia‐Pagan et al. (1996), moderate exercise reduces HS blood flow in patients with cirrhosis. Accordingly at vigorous intensity, HS blood flow was reduced to approximately falf of the resting level, and it reached its lowest value in the immediate recovery from exercise. The reduction in HS blood flow was established at a lower heart rate than during exercise in healthy subjects (Eriksson et al., 1985; Nielsen et al., 2002) but heart rate was raised to a higher level than previously reported in cirrhotic patients (Garcia‐Pagan et al., 1996). While HS blood flow was reduced by ~34%, a more dramatic decrease of ~60% was reported in healthy subjects under similar experimental conditions (Nielsen et al., 2002). In that study, exhaustion was associated with signs of sinusoidal collapse with hepatic venous oxygen saturation as low as 6%. The more pronounced HS blood flow reduction in the healthy subjects is likely related to their aerobic fitness‐related higher sympathetic activation capacity compared to the less fit cirrhotic patients (Kjaer, 1998). Beta‐adrenergic receptor blockade affects HS blood flow (Ahlborg & Juhlin‐Dannfelt, 1994) as cardiac output becomes reduced (Pawelczyk et al., 1992), but oral beta‐adrenergic blocker treatment was discontinued and exercise reduced HS blood flow in the two beta‐blocker treated patients similarly to those not on beta‐blockers, by 46% and 35%, respectively.

### 4.3 Glucose flux

The increased HS glucose output observed during vigorous exercise (from ~0.5 to ~1.5 mmol/min) suggests that endogenous glucose production was maintained during exercise and increased to almost comparable level (~2.4 mmol/min) with healthy people under similar conditions (Nielsen et al., 2002). Our findings are in disparity with the report of compromised endogenous glucose production in four patients with cirrhosis during treadmill exercise (De Lissio et al., 1991). However, in that report liver venous concentrations were not measured, hypoglycemia was not observed during exercise, and it was speculated that muscle metabolism shifted to fatty acids since they were increased in peripheral blood. In contrast, in the present study HS uptake and arterial FFA levels were reduced (Table 2), while euglycemia was maintained during both exercise and recovery, and thus was not associated with an increased reliance on fat metabolism. Using direct measurements in the hepatic vein, we demonstrated that glucose production increased in a stepwise manner in response to exercise (Figure 2). The direct methodology applied allows the conclusion that endogenous glucose production is maintained during exercise, at least in patients in Child Pugh class B cirrhosis.

### 4.4 Lactate flux

The progressive HS lactate uptake observed during exercise in our study, together with the clearance half‐time during recovery (~15 min), do not support decreased hepatic lactate clearance following maximal exercise in cirrhotic patients (Almenoff et al., 1989). Specifically, the half‐time is comparable with that reported for healthy subjects in the abovementioned study and in the study by Eriksson et al (1985). In support, HS lactate clearance has been reported to be higher in fasting and resting patients with cirrhosis than in healthy subjects (Jeppesen et al., 2013). As two lactate molecules are required to synthesize glucose, the HS removal of lactate could quantitatively account for approximately two‐thirds of the glucose production (Figures 2 and 3). Furthermore, since the arterial lactate values at the end of the vigorous exercise trial were high (~9 mM), it is unlikely that the relatively short lactate clearance half‐time was due to low maximal concentration values. The discrepancy between our findings and those of Almenoff et al. (1989) may be related to the different disease severity of the patients investigated since most of them had ascites (Jeppesen et al., 2013). Furthermore, in healthy persons during similar conditions, hepatic lactate removal also increased during exercise but at near exhaustion, the HS perfusion was reduced to a degree where lactate clearance abruptly decreased (Nielsen et al., 2002). This circulatory collapse suggests that the reduction of lactate clearance was a manifestation of the reduced HS blood flow rather than exhaustion of the glyconeogenetic capacity of the healthy liver. A similar collapse of HS perfusion was not observed in patients with cirrhosis in the present study. Thus, we conclude that, at least in patients with Child B cirrhosis, the contribution of HS lactate uptake to lactate metabolism during exercise is maintained and, therefore, is not solely responsible for the development of lactacidemia during vigorous exercise.

### 4.5 Ammonia flux

The almost three‐fold increase of HS ammonia uptake compared to rest does not support reduced hepatic ammonia clearance, consequent to impaired hepatic function (Dietrich et al., 1990). Despite the elevated hepatic clearance, hyperammonemia was not prevented during vigorous exercise, as arterial ammonia reached even higher levels than reported for cirrhotic patients (144 vs. 124 μM) (Dietrich et al., 1990). However, considering that exercise, at comparable relative intensity and duration in healthy subjects provokes a similar elevation in plasma ammonia (127 μM) (Babij et al., 1983), or even higher during prolonged exercise (190 μM) (Nybo et al., 2005), the present findings do not allow to conclude that the hyperammonemia observed is attributed entirely to compromised hepatic ammonia clearance due to cirrhosis. This is in accordance with the observation that elevated ammonia in resting patients with cirrhosis is primarily due to enhanced production and systemic ammonia clearance is only moderately reduced by 20% (Eriksen et al., 2023).

### 4.6 General considerations

Cirrhosis is characterized by reduced hepatocellular function as well as altered hepatic perfusion where fibrosis leads to increased resistance, portal hypertension with development of porto‐systemic collaterals, and “arterialization” as hepatic arterial perfusion gradually increases from a normal value of 1/3 up to 100%. Even though a definitive quantitative comparison of metabolic responses to exercise in cirrhotic and healthy subjects would require the inclusion of healthy controls performing the same exercise protocol as that of the present study, nevertheless, a qualitative interpretation of the present data improves understanding of hepatic metabolism in cirrhosis during exercise.

In the present study, the hepatic metabolic response to exercise was qualitatively different from that of healthy subjects: increased lactate uptake could account for approximately 2/3 of the glucose output. Metabolism of amino acids and FFA could quantitatively explain the remaining 1/3, so exhaustion of the glycogen pool or impaired glycogenolysis was unlikely. This is supported by a report of 28 g (155 mmol) glycogen per liver in patients with cirrhosis (Krähenbühl et al., 2003), which is well above the accumulated glucose output in the present study. An eventual evaluation of the effect of glycogen storage exhaustion would require an exercise protocol of 2–3 h. At the same time, lactate concentrations reached higher levels (~9 mmol/L) than in healthy persons exercising at a higher workload (~6–8 mmol/L) (Eriksson et al., 1985; Nielsen et al., 2002), indicating that the muscular production of lactate during exercise may be higher in patients, or that the total capacity of this metabolic function is quantitatively reduced in cirrhosis, although qualitatively normal.

At rest, muscles remove ammonia from the blood stream by converting glutamate to glutamine, which is subsequently released into the bloodstream. During exercise, this situation is reversed and muscles release ammonium from degradation of AMP to IMP (Katz et al., 1986). Similar to other studies (Eriksson et al., 1985; Katz et al., 1986), lactate and ammonia increased in parallel, as would be expected since the production of both is linked to insufficient energy production relative to energy demands. The HS metabolism of ammonia is complex, and our study was limited by its inability to sample portal blood. As the intestines metabolize glutamine to glutamate and ammonium as an energy source, portal blood contains lower glutamine and higher glutamate levels relative to arterial blood. In the liver, glutamate and ammonia are taken up for the production of glutamine, which is released in sinusoidal blood, while at the same time glutamine is removed by the formation of urea. The net result is that the liver removes glutamate and releases glutamine, as observed in this study. The fact that the HS release of glutamate decreased during exercise (Table 2) likely reflects a reduction of the intestinal perfusion. Apart from that, hepatic handling of ammonia was qualitatively normal. While ammonia clearance is moderately reduced by 20% in patients with cirrhosis (Eriksen et al., 2023), increased muscular production of ammonia during exercise in patients with cirrhosis may be also considered. Although vigorous exercise in healthy subjects may lead to a degree of liver circulatory collapse that constrains lactate removal (Nielsen et al., 2002), a similar phenomenon was not observed in our patients with cirrhosis. We speculate that arterialization of cirrhotic liver perfusion may have prevented such circulatory collapse, even though the portal contribution decreased during exercise due to intestinal and splenic arterial contractions. A similar supportive role of the hepatic artery to hepatic circulation was observed during bleeding, where hepatic arterial flow was maintained but portal venous flow was reduced (Rasmussen et al., 1999).

### 4.7 Limitations

The patients had different backgrounds for the development of cirrhosis, which may have affected the results. Ideally, the inclusion of a healthy control group would have allowed direct comparison instead of referring to previous studies on healthy subjects using a similar exercise protocol (Nielsen et al., 2002). However, due to the invasive procedures used during cycling a control group was not included and only a small number of patients participated. We recognize that an evaluation of liver blood flow by ICG does not allow for a distinction from at least three different elements: the hepatic artery flow, the flow in the portal vein from the intestine, and the part of the portal flow originating from the spleen. As demonstrated by Perko et al. (1998) and confirmed in women (Endo et al., 2008), a reduction in portal blood flow may be compensated for by the hepatic artery, as also demonstrated in pigs during hemorrhage (Rasmussen et al., 1999).

In conclusion, euglycemia was maintained during vigorous exercise, but the liver failed to prevent arterial accumulation of lactate and ammonia, which may contribute to the development of lactacidemia‐ and hyperammonemia that exacerbate fatigue and exercise intolerance in cirrhotic patients.

## AUTHOR CONTRIBUTIONS

Study concept and design: SV, NHS, OC, PO, and HBN; acquisition of data: SV, NHS, OC, PO, and HBN; analysis and interpretation of data: SV, NHS, OC, PO, and HBN; drafting of the manuscript: SV, NHS, OC, PO, and HBN; critical revision of the manuscript for important intellectual content: SV, NHS, OC, PO, and HBN.

## FUNDING INFORMATION

No funding information provided.

## CONFLICT OF INTEREST STATEMENT

None.

## Data Availability

Original data are available upon reasonable request.
